# Pathological pain processing in mouse models of multiple sclerosis and spinal cord injury: contribution of plasma membrane calcium ATPase 2 (PMCA2)

**DOI:** 10.1186/s12974-019-1585-2

**Published:** 2019-11-08

**Authors:** Ersilia Mirabelli, Li Ni, Lun Li, Cigdem Acioglu, Robert F. Heary, Stella Elkabes

**Affiliations:** 1 The Reynolds Family Spine Laboratory, Department of Neurosurgery, New Jersey Medical School, Rutgers, The State University of New Jersey, Newark, NJ 07103 USA; 2 School of Graduate Studies, New Jersey Medical School, Rutgers, The State University of New Jersey, Newark, NJ 07103 USA

**Keywords:** Neuropathic pain, Calcium, ATP2b2, Multiple sclerosis, Spinal cord injury, Cytokine, Nociception, Inflammation

## Abstract

**Background:**

Neuropathic pain is often observed in individuals with multiple sclerosis (MS) and spinal cord injury (SCI) and is not adequately alleviated by current pharmacotherapies. A better understanding of underlying mechanisms could facilitate the discovery of novel targets for therapeutic interventions. We previously reported that decreased plasma membrane calcium ATPase 2 (PMCA2) expression in the dorsal horn (DH) of healthy PMCA2^+/−^ mice is paralleled by increased sensitivity to evoked nociceptive pain. These studies suggested that PMCA2, a calcium extrusion pump expressed in spinal cord neurons, plays a role in pain mechanisms. However, the contribution of PMCA2 to neuropathic pain processing remains undefined. The present studies investigated the role of PMCA2 in neuropathic pain processing in the DH of wild-type mice affected by experimental autoimmune encephalomyelitis (EAE), an animal model of MS, and following SCI.

**Methods:**

EAE was induced in female and male C57Bl/6N mice via inoculation with myelin oligodendrocyte glycoprotein fragment 35–55 (MOG_35–55_) emulsified in Complete Freund’s Adjuvant (CFA). CFA-inoculated mice were used as controls. A severe SC contusion injury was induced at thoracic (T8) level in female C57Bl/6N mice. Pain was evaluated by the Hargreaves and von Frey filament tests. PMCA2 levels in the lumbar DH were analyzed by Western blotting. The effectors that decrease PMCA2 expression were identified in SC neuronal cultures.

**Results:**

Increased pain in EAE and SCI was paralleled by a significant decrease in PMCA2 levels in the DH. In contrast, PMCA2 levels remained unaltered in the DH of mice with EAE that manifested motor deficits but not increased pain. Interleukin-1β (IL-1β), tumor necrosis factor α (TNFα), and IL-6 expression were robustly increased in the DH of mice with EAE manifesting pain, whereas these cytokines showed a modest increase or no change in mice with EAE in the absence of pain. Only IL-1β decreased PMCA2 levels in pure SC neuronal cultures through direct actions.

**Conclusions:**

PMCA2 is a contributor to neuropathic pain mechanisms in the DH. A decrease in PMCA2 in DH neurons is paralleled by increased pain sensitivity, most likely through perturbations in calcium signaling. Interleukin-1β is one of the effectors that downregulates PMCA2 by acting directly on neurons.

## Background

Neuropathic pain, a complex and chronic condition, is often associated with various pathologies including multiple sclerosis (MS) [1] and spinal cord injury (SCI) [2]. It remains the most challenging type of pain to treat, since conventional therapies are frequently ineffective.

Multiple sclerosis is an inflammatory, demyelinating disease of the central nervous system (CNS), whose precise etiology is still unknown [3]. Although the disease has been primarily characterized by motor deficits, chronic pain is a disabling symptom experienced by 60% of MS patients [4, 5]. Chronic pain develops secondary to demyelination, neuroinflammation, and axonal damage in the CNS [6]. Similarly, chronic pain is common among individuals sustaining a SCI and affects quality of life [7]. The complex and vast cascade of events that follows spinal cord (SC) trauma causes pathological alterations at the lesion site but also in regions remote from the injury epicenter and rostral or caudal to the lesion site [8]. This can lead to neuropathic pain originating above, below, or at the level of the injury [9]. Evidence suggests that hyperactivation of second order sensory neurons in the dorsal horn (DH) of the SC is an important mechanism underlying neuropathic pain [10]. Effectors released by activated glia and/or infiltrating immune system cells, especially cytokines, including interleukin-1β (IL-1β), IL-6, and tumor necrosis factor α (TNFα) have been implicated in the hyperactivation of DH neurons [11, 12]. Despite many advances, the mechanisms underlying neuropathic pain have not been fully characterized and the molecular events that result in the hyperactivation of neurons are inadequately defined. Therefore, further investigations are needed to identify novel targets that contribute to pain mechanisms. The discovery of new targets could facilitate the design of therapeutic approaches that alleviate neuropathic pain more effectively.

Recent investigations in our laboratory indicated that plasma membrane calcium ATPase 2 (PMCA2) could play an important new role in mechanisms of pain processing in the DH of the SC [13, 14]. PMCA2 belongs to a family of calcium extrusion pumps present in a variety of cells [15–17]. Various PMCA isoforms participate in the regulation of intracellular calcium levels during physiological and pathological conditions [18–21]. Four PMCA isoforms have been described: PMCA1 and 4 are expressed ubiquitously, whereas the distributions of PMCA2 and PMCA3 are more restricted [22, 23]. In the CNS, PMCA2 and PMCA3 are primarily expressed in neurons [24, 25]. PMCA2 is also expressed in DH neurons, which receive pain messages from the dorsal root ganglia (DRG) and convey them to the brain [24].

Earlier reports from our laboratory demonstrated that adult, female PMCA2-heterozygous (PMCA2^+/−^) mice show increased mechanical pain sensitivity when compared to female, wild-type (PMCA2^+/+^) littermates [13]. Furthermore, lower PMCA2 expression in the DH of female PMCA2^+/−^ mice was paralleled by specific changes in the expression of select glutamate receptors implicated in pain processing [13]. Since PMCA2 expression was not detected in the DRG, where primary sensory neurons reside, the findings led to the postulate that reduced PMCA2 expression in the DH corresponds with increased mechanical pain sensitivity. It is important to note that these previous studies assessed evoked pain responses in genetically modified mice that did not sustain injury or were not affected by disease. As the involvement of PMCA2 in DH pain mechanisms during injury or disease has not been investigated, studies were undertaken to examine the role of PMCA2 in pathological pain using two distinct mouse models of injury or disease. One of these animal models is experimental autoimmune encephalomyelitis (EAE), a clinically relevant mouse model that mimics MS pathology [26]. Although EAE is primarily characterized by progressive motor deficits leading to paralysis, increased pain sensitivity has also been observed [27, 28]. The second is a murine SC contusion injury model, which causes paralysis and chronic pain [29]. We assessed whether the manifestation of pain in EAE and SCI is associated with a reduction in PMCA2 levels in the DH, and we identified the triggers that decrease PMCA2 expression in SC neurons, in vitro.

## Methods

### Animals

Eight-week-old female or male C57Bl/6N (C57Bl/6NTac; Taconic Biosciences, Rensselaer, NY) and C57Bl/6Ncrl mice (Charles River Laboratories, Wilmington, MA) were housed in a pathogen-free barrier facility and maintained on a 12-h dark/light cycle with food and water given ad libitum. Sentinels were housed in the same room and periodically checked for infection.

### Induction of EAE

Eight-week-old C57Bl/6NTac or C57Bl/6Ncrl mice were inoculated subcutaneously (sc) in the flank with 100 μg of myelin oligodendrocyte glycoprotein fragment 35–55 (MOG_35–55_; MEVGWYRSPFSRVVHLYRNGK; Stanford PAN Facility, Stanford, CA, USA) emulsified in Complete Freund’s Adjuvant (CFA, Becton, Dickinson and Company, Franklin Lakes, NJ, USA) containing *Mycobacterium tuberculosis* H37RA (7 mg/ml MT, Difco Laboratories, Detroit, MI, USA). Control mice were inoculated with CFA/vehicle. Both groups received intraperitoneal (ip) injections of pertussis toxin (0.3 μg/mouse, List, Biological Laboratories Inc., Campbell, CA, USA) on days 1 and 3 post-inoculation with MOG_35–55_/CFA. Motor symptoms were scored starting on day 8 post-inoculation. Weakness of the distant part of the tail was considered as the first motor deficit. A clinical scale that ranged from 0 to 5 was used to evaluate motor symptoms as follows: 0, no symptoms; 0.5, weakness of the distant part of the tail; 1, flaccid tail; 2, hindlimb paresis; 3, hindlimb paralysis; 4, hindlimb and forelimb paralysis; and 5, moribund or dead. Pain sensitivity was always assessed when mice exhibited a flaccid tail (clinical score 1). Thereafter, mice were euthanized by exposure to CO_2_ to collect the lumbar DH tissue. For comparative studies, C57Bl/6NTac or C57Bl/6Ncrl mice were inoculated in parallel with the same MOG_35–55_ or CFA preparation and evaluated in behavioral tests concomitantly.

### Induction of SCI

Eight-week-old, female C57Bl/6NTac mice were anesthetized with ketamine (80 mg/kg; Vedco, St. Joseph, MO, USA) and xylazine (10 mg/kg; Akorn Inc., Decatur, IL, USA) that was administered via an ip injection. Following a laminectomy at thoracic vertebral level 8 (T8), a severe contusion injury (70 kilodyne force) was induced using the Infinite Horizon Impactor (Precision Systems and Instrumentation, Lexington, KY, USA). Sham mice were anesthetized and received a laminectomy at T8. Uninjured mice were anesthetized, and a skin incision was performed. The incision was closed with wound clips to ensure blinded evaluations. Immediately postoperatively, all mice received subcutaneous (sc) injections of Lactated Ringers solution (1 ml; Baxter, Deerfield, IL, USA), Baytril (2.5 mg/kg; Bayer, Kansas City, KS, USA), and Buprenorphine (75 μg/kg; Hospira, Lake Forest, IL, USA). Antibiotics, analgesics, and 1 ml of normal saline (Baxter) were administered to mice twice daily for 7 days. Bladders were manually expressed twice per day. On 1, 7, 14, 21, and 28 days post-injury (dpi), hindlimb locomotor function was assessed using the Basso Mouse Scale (BMS) [30] by two experimenters who were blinded to the experimental arms. Pain sensitivity was evaluated on 28 dpi.

### Behavioral analyses for the assessment of pain

The Hargreaves’ plantar thermal test and the von Frey filament test were performed by a single evaluator blinded to the experimental conditions.

#### Plantar thermal test (Hargreaves’ method)

Mice were habituated for 30 min to restrainers placed on a glass surface (IITC Life Science, Woodland Hills, CA, USA). A beam of light with intensity of 15 was delivered to the plantar surface of the hindpaw. The paw withdrawal latency was recorded five times for each hindpaw and then averaged, with the final value being the average of the right and left hindpaw averages. The cutoff time for observation was set to 20 s to avoid tissue damage.

#### von Frey filament test

Mice were habituated to the plexiglass observation chambers 1 h daily for 2 consecutive days and for 30 min on the day of the test. The paw withdrawal threshold to a set of von Frey filaments (ranging from 0.008 to 2.0 g; North Coast Medical, Morgan Hill, CA, USA) was measured on the left and right hindpaws using the up-down paradigm as described previously [13]. The average of the left and right hindpaw thresholds was calculated as the final value.

### Tissue collection

Immediately after euthanasia by CO_2_ inhalation, mice were decapitated. The SCs were removed, and the DH was dissected. The tissue was frozen on dry ice and stored at − 80 °C until use.

### Western blot analysis

The lumbar DH tissue was homogenized with a motorized pestle in 100 μl of ice-cold lysis buffer that contained 10 mM 4-(2-hydroxyethyl)-1-piperazineethanesulfonic acid (HEPES) buffer, 10% sucrose, and 5 mM ethylenediaminetetraacetic acid (EDTA) at pH 7 in the presence of a protease inhibitor cocktail (1:100; Sigma-Aldrich, St. Louis, MO, USA) freshly added to the lysis buffer. The homogenates were centrifuged at 800 relative centrifugal force (rcf) for 5 min to pellet the nuclei. The supernatant was centrifuged again at 20,000 rcf for 45 min to separate soluble proteins from membrane-bound proteins. The pellet containing membranes was then re-suspended in Universal Protein Extraction (UPX, Expedeon, San Diego, CA, USA) lysis buffer, frozen in a slurry of 2-methylbutane mixed with dry ice and thawed at 37 °C. The freeze/thaw cycle was repeated 4 times and the samples were then mixed for 15 s by use of a vortex and then incubated on ice for 15 s. These steps were repeated for a total of 5 times. Subsequently, the suspension was sonicated for 30 s in a low-frequency ultrasonic bath and incubated on ice for 30 min. The concentrations of the soluble proteins and those extracted from membranes were quantified using the Bio-Rad DC (Detergent compatible; Bio-Rad, Hercules, CA, USA) protein assay according to the manufacturer’s instructions.

Five to 10 μg of protein were loaded in each lane of a 4–15% stain-free Tris-Glycine eXtended (TGX, Bio-Rad) gel, and electrophoresis was performed for 45 min at 200 V. Proteins were then electrotransferred to polyvinylidene difluoride membranes (PVDF, Bio-Rad) for 6 or 15 min, depending on the molecular weight of the protein of interest, at 25 V using the Trans-Blot Turbo transfer system (Bio-Rad). Membranes were then blocked 1 h in 5% non-fat dry milk powder dissolved in Tris-buffered saline (TBS—20 mM Trizma base and 500 mM NaCl, pH 7.4) containing 0.1% of Tween-20 (T-TBS) and incubated overnight with a primary antibody. The membranes were probed with antibodies against PMCA2 (1:20000, Swant Inc., Marly, Switzerland), PMCA3 (1:5000, Abcam, Cambridge, MA, USA), PMCA4 (1:5000, Abcam), Glial fibrillary acidic protein (GFAP, 1:5000, Abcam), and ionized calcium binding adaptor molecule 1 (Iba1, 1:1000, Wako Chemicals, Richmond, VA, USA) followed by secondary antibodies horseradish peroxidase (HRP)-conjugated polyclonal donkey anti-rabbit IgG or unconjugated polyclonal goat anti-mouse IgG + IgM (Jackson ImmunoResearch Laboratories, Inc., West Grove, PA, USA). Signals were detected using Clarity Western ECL Substrate (Bio-Rad) on the ChemiDoc Touch Imaging System (Bio-Rad). Quantification of band densities was performed using Image J software (National Institute of Health, Bethesda, MD, USA) and normalized to total protein which is determined by measuring and adding the intensity of different molecular weight bands.

### Quantitative reverse transcription-polymerase chain reaction (qRT-PCR)

Total RNA was isolated from the lumbar DH as described previously [29]. Five hundred nanograms of total RNA was treated with ezDNAse (Thermo Fisher Scientific Inc., Waltham, MA, USA) for 2 min at 37 °C to digest residual genomic DNA and subsequently reverse transcribed using SuperScript™ IV VILO™ Master Mix (Thermo Fisher Scientific Inc.). Quantitative PCR was performed using PowerUp™ SYBR™ Green Master Mix (Thermo Fisher Scientific Inc.). Samples were heated at 95 °C for 2 min and amplified for 40 cycles with denaturation at 95 °C for 15 s, annealing at 60 °C for 15 s, and extension at 72 °C for 1 min. The threshold cycle number (C_T_) of each target was calculated and expressed relative to that of glyceraldehyde 3-phosphate dehydrogenase (GAPDH), and the ΔΔC_T_ values of targets were then calculated and presented as relative fold induction. All primers were used at 0.1 μM concentration and were purchased from Real Time Primers (Elkins Park, PA, USA). In some of the experiments, the RT-PCR products were resolved on an agarose gel, which was run at 140 V for 1 h.

### Flow cytometry

Mice that reached the clinical score of 1 were anesthetized with ketamine/xylazine (ip). They were then perfused with saline. The lumbar DH was dissected and manually minced in Hank’s Balanced Salt Solution (HBSS; Thermo Fisher Scientific Inc.) treated with trypsin (0.5 mg/ml; Thermo Fisher Scientific Inc.) and collagenase (1 mg/ml; Sigma-Aldrich) in Dulbecco’s modified Eagle’s medium (DMEM; Thermo Fisher Scientific Inc.), passed through a 40-μm strainer, and re-suspended in ACK lysing buffer (Quality Biological Inc., Gaithersburg, MD, USA). Following washes with Dulbecco’s phosphate-buffered saline (DPBS; Corning, Corning, NY, USA) and fluorescence-activated cell sorting (FACS) buffer (DPBS containing 2% fetal bovine serum (FBS) and 0.09% sodium azide), the samples were incubated first with Fc Block (BD Bioscience, Franklin Lakes, NJ, USA) for 10 min and then with an antibody against CD45 (1:100, BD Bioscience) or isotype control (1:100, BD Bioscience) in FACS buffer for 30 min and fixed with 4% paraformaldehyde in phosphate-buffered saline, pH 7.5 (PF/PBS). Sample acquisition was performed using the BD LSRII Flow Cytometer, and the data were analyzed using FlowJo software (V 10.0.8; Tree Star Inc., Ashland, OR, USA).

### Immunohistochemistry (IHC)

Mice were anesthetized with a mixture of ketamine/xylazine followed by transcardial perfusion with saline. Spinal cords were removed and quickly embedded in optimal cutting temperature (OCT) compound and frozen in a dry ice-ethanol mix. Thirty-micrometer-thick transverse lumbar SC sections were obtained on a cryostat and mounted on slides. The sections were first fixed in cold methanol for 10 min and then blocked with 30% normal goat serum (NGS) in 10 mM phosphate-buffered saline (PBS, pH = 7.4) for 1 h at room temperature (RT) followed by overnight incubation in rabbit polyclonal antibody against PMCA2 (1:1000; Swant) at 4 °C. After PBS rinses, the sections were incubated with a goat anti-rabbit Alexa Fluor 488 secondary antibody (1:500; Thermo Fisher Scientific Inc.) for 1 h at RT, and followed by an overnight incubation with a mouse monoclonal antibody against NeuN (1:500; Millipore Sigma) at 4 °C, and finally probed with a goat anti-mouse Alexa Fluor 594 secondary antibody (1:500; Thermo Fisher Scientific Inc.) for 1 h at RT. The sections were then coverslipped with ProLong Diamond antifade mountant containing DAPI (Thermo Fisher Scientific Inc.).

For immunolabeling of astrocytes and microglia, mice were anesthetized and euthanized by transcardial perfusion with saline followed by 4% paraformaldehyde in 0.1 M phosphate buffer (PFA, pH = 7.4). The spinal cords were dissected and post-fixed in 4% PFA overnight and cryoprotected in 27% sucrose/PBS for 24 h before being embedded in OCT. Thirty micrometer transverse cryostat sections were blocked in 30% NGS containing 0.1% Triton X-100 and then probed overnight with either rabbit polyclonal antibodies against GFAP (1:1000, Agilent Technologies, Santa Clara, CA, USA), an astrocyte marker, or rabbit polyclonal antibodies against Iba1 (1:500; Wako Chemicals, Richmond, VA, USA), a microglia/macrophage marker, diluted in PBS containing 0.1% Triton X-100. The sections were then incubated with a goat anti-rabbit Alexa Fluor 488 secondary antibody (1:500; Thermo Fisher Scientific Inc) and coverslipped with ProLong Diamond antifade mountant containing DAPI (Thermo Fisher Scientific Inc.). Fluorescent images were captured on a Nikon A1R confocal microscope with a × 60 objective using NIS Elements AR 4.00.07 software.

### Histological approaches

Luxol fast blue staining was used to visualize myelin. Transverse SC cryostat sections were dehydrated by use of 85%, 95%, and 100% ethanol followed by xylene. They were incubated in 0.1% Luxol fast blue overnight at 56 °C (Thermo Fisher Scientific Inc.). The next day, the sections were differentiated in 0.05% lithium carbonate (Sigma Aldrich) and 70% ethanol, dehydrated with increasing concentrations of ethanol, and coverslipped using Permount (Thermo Fisher Scientific Inc.) mounting media. Pictures were obtained using a Nikon Eclipse 50i microscope equipped with a × 4 objective, using Ocular software (Digital Optics Ltd., Auckland, New Zealand).

Hematoxylin and eosin staining was performed with an automated stainer (Leica Autostainer XL, Nussloch, Germany). The SC sections were immersed in Harris hematoxylin and eosin stain. Following dehydration in 95% and 100% alcohol solutions, the section were coverslipped using Permount mounting media (Thermo Fisher Scientific Inc.) and pictures were taken with a Nikon Eclipse 50i microscope equipped with a × 10 objective and using the Ocular software (Digital Optics Ltd).

### Stereological cell counts

PMCA2/NeuN double-labeled and total NeuN positive cell counts were obtained using the Optical Fractionator probe of Stereo Investigator V.11.11.2 (MBF Bioscience, Williston, VT, USA) and an Olympus BX51 microscope (Olympus Inc., Melville, NY, USA) with a × 40 objective. The number of labeled cells was estimated based on planimetric volume calculations in Stereo Investigator with a counting frame of 75 μm by 75 μm, a dissector height of 15 μm, and the guard zones of 2 μm. A total of 3 sections at 120 μm intervals were counted per lumbar SC, and the estimated cell density in the DH was reported per cubic millimeter.

### Spinal cord neuronal cultures

Timed pregnant C57Bl/6NTac mice on gestation day 13 were euthanized by exposure to CO_2_. The embryos were removed, and the SCs were dissected. The SC cell cultures were prepared as described previously [31]. Shortly, the tissue was dissociated by trituration and the suspension was layered on a 4% bovine serum albumin (BSA) gradient and centrifuged at 1500 rpm for 2 min. The pellet containing the dissociated cells was re-suspended in neurobasal medium containing 2% N-21 supplements (Miltenyi Biotec, Somerville, MA, USA), 6.3 mg/ml NaCl, and 10 units/ml penicillin/streptomycin (NBM^+^) and plated in poly-l-ornithine-coated (Sigma-Aldrich) 35 mm dishes at a density of 6 × 10^5^ cells/dish. Half of the media was replaced with fresh NBM^+^ every 72 h. The neuronal cultures were maintained for 11 days in a humidified incubator with 5% CO_2_ and 95% air, at 37 °C to allow for maturation. Media were purchased from Fisher Thermo Scientific.

### Treatment of EAE mice with IL-1RA

EAE was induced in C57Bl/6NTac mice as described above. At the time when the tip of the tail showed weakness (clinical score 0.5), recombinant mouse IL-1ra/IL-1F3 protein (IL-1RA, R&D Systems, Inc., 20 ng/g body weight) or vehicle (sterile PBS with 0.1% BSA) was administered by lumbar puncture. A second intrathecal injection was given 24 h later. Pain was assessed when flaccid tail was observed as described above.

### Statistical analyses

GraphPad statistical package was used for all analyses. A two-tailed independent sample *t* test with a Bonferroni correction or a one-way analysis of variance (ANOVA) followed by Tukey’s honestly significant difference (HSD) post hoc test was performed. Data are presented as the mean ± standard error of the mean (SEM). A *p* value < 0.05 was considered to be statistically different.

## Results

### PMCA2 levels are decreased in the DH of C57Bl/6NTac mice manifesting increased pain sensitivity during EAE

**Fig. 1:**
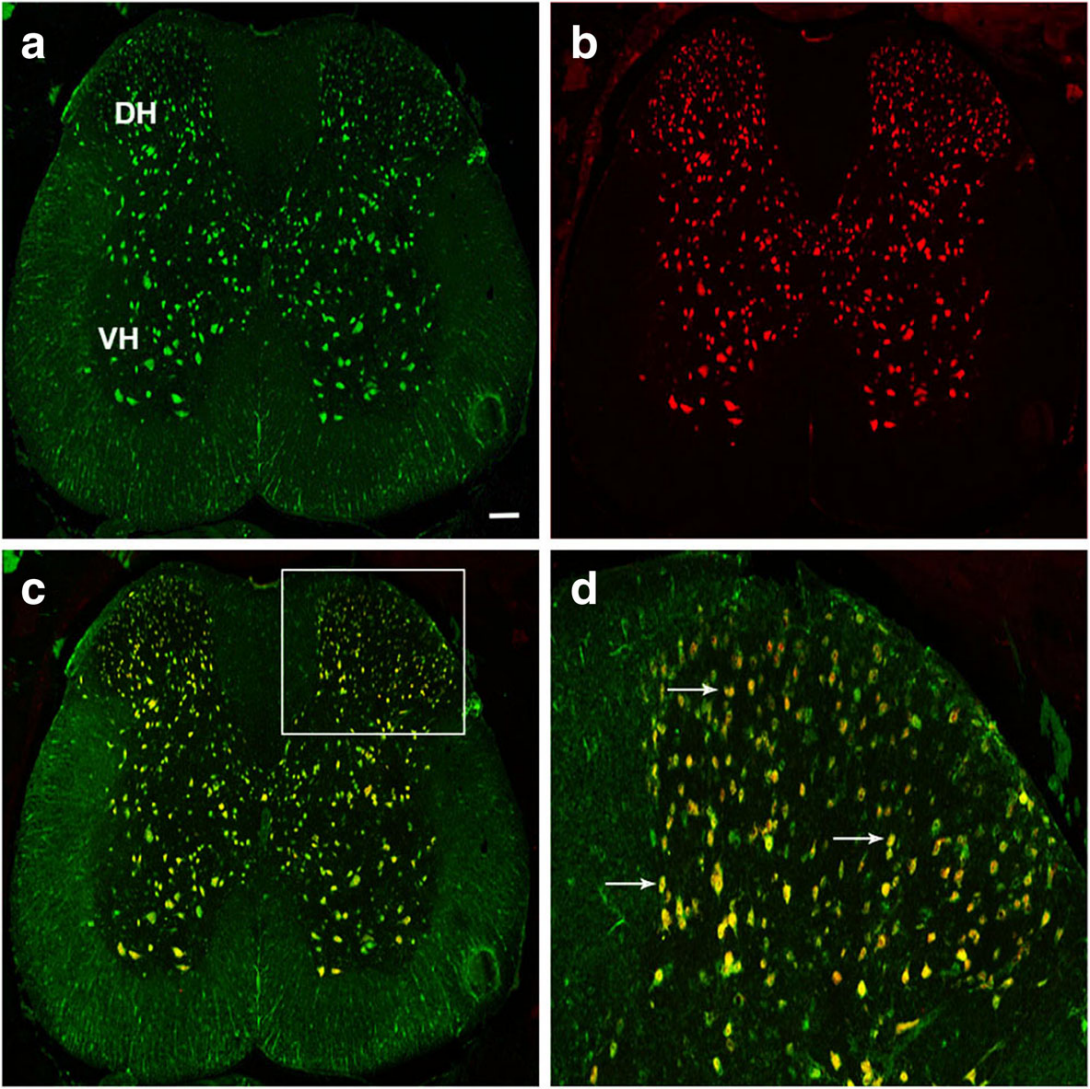
Co-localization of PMCA2 and NeuN in the DH of the mouse SC. A representative transverse lumbar SC section showing **a** PMCA2 immunoreactive cells, **b** NeuN immunoreactive cells, and **c** PMCA2/NeuN double-labeled cells. **d** A higher magnification picture of the area delineated by the square in **c**. Arrows point at examples of PMCA2/NeuN double-labeled cells. Scale bar represents 100 μm in **a**–**c**

We first confirmed that PMCA2 protein is expressed in neurons of the mouse SC (Fig. 1). PMCA2 immunoreactivity (Fig. 1a) was co-localized with NeuN (Fig. 1b), a neuronal marker, throughout the grey matter of the SC, including the DH (Fig. 1c, d). In contrast, PMCA2 immunoreactive cell bodies were not found in the white matter (Fig. 1a). The co-localization and distribution of PMCA2 and NeuN suggested that both projection neurons and interneurons express PMCA2.

**Fig. 2:**
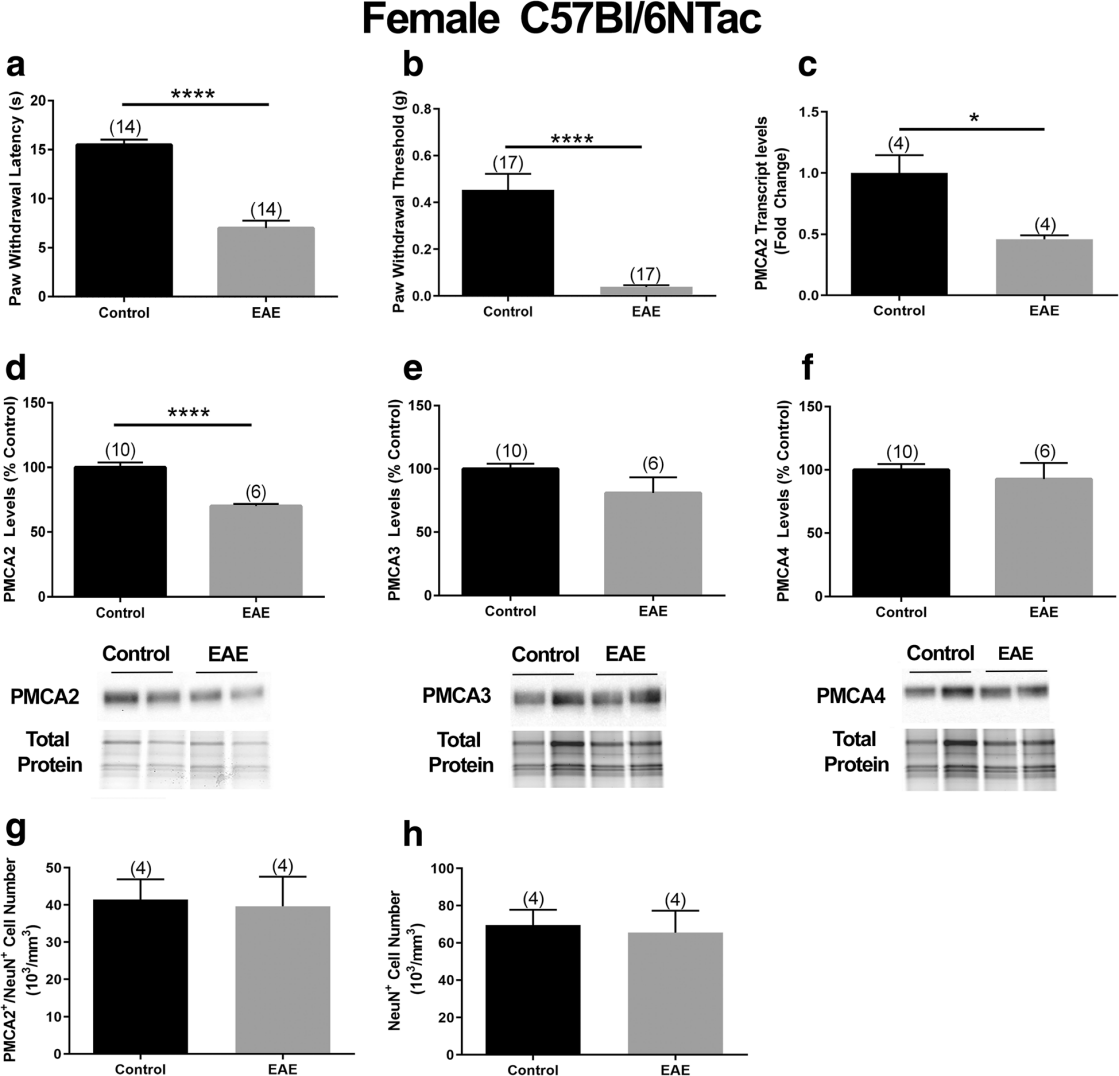
PMCA2 levels in the lumbar DH of female C57Bl/6NTac mice with EAE manifesting pain. Evaluation of thermal (heat) and mechanical pain sensitivity in female mice at early phase of EAE by the **a** Hargreaves’ test and **b** von Frey filament test. **c** PMCA2 transcript levels in the lumbar DH. **d**–**f** PMCA2, PMAC3, and PMCA4 protein levels in the lumbar DH. The graphs (upper panel) show the quantification of the band intensity in the Western blots (lower panel; two representative lanes per group). Total protein was used to normalize for experimental variations. **g** Stereological quantification of PMCA2/NeuN double-labeled neurons in the lumbar DH. **h** Total NeuN immunoreactive cell number in the lumbar DH. The number of mice in each group is shown above bars. Values represent mean ± SEM. Significantly different by independent Student’s *t* test with *p < 0.05 and ****p < 0.0001

Subsequently, we investigated a potential link between PMCA2 expression and increased pain sensitivity in EAE using the female C57Bl/6NTac mice. Our goal was to first determine whether mice that display motor deficits in EAE also manifest heightened pain sensitivity, and then assess whether PMCA2 levels in the DH of these mice are altered. We focused on the early disease phase, the time when only a flaccid tail (clinical score 1) is observed, because at subsequent disease phases, hindlimb paresis and paralysis interfere with the assessment of pain sensitivity. The von Frey filament and the Hargreaves paw withdrawal tests were used to evaluate mechanical and thermal pain, respectively. All female C57Bl/6NTac mice exhibiting a flaccid tail also displayed increased pain sensitivity. There was a 55% decrease in paw withdrawal latency in the Hargreaves test (Fig. 2a) and an 85% decrease in the paw withdrawal thresholds in the von Frey filament test (Fig. 2b) in mice with EAE as compared to the CFA-inoculated mice used as controls. Next, we determined the PMCA2 levels in the DH of these mice. There was a significant 50% reduction in PMCA2 transcript levels (Fig. 2c) and a 35% reduction in PMCA2 protein levels (Fig. 2d) in the DH of mice with EAE compared to CFA-inoculated controls. The decrease in PMCA2 levels was selective since the protein levels of two other PMCA isoforms, PMCA3 and PMCA4, remained unaltered (Fig. 2e, f). To ensure that the decrease in PMCA2 is not due to cell loss, the number of PMCA2/NeuN double-labeled neurons as well as total NeuN immunoreactive neurons in the lumbar DH was quantified by stereological cell counting. There were no statistical differences in the number of PMCA2/NeuN or total NeuN immunoreactive neurons in the lumbar DH of EAE mice compared to CFA-inoculated controls (Fig. 2g, h) confirming that the decrease in PMCA2 is not due to the loss of PMCA2 expressing DH neurons in EAE mice.

**Fig. 3:**
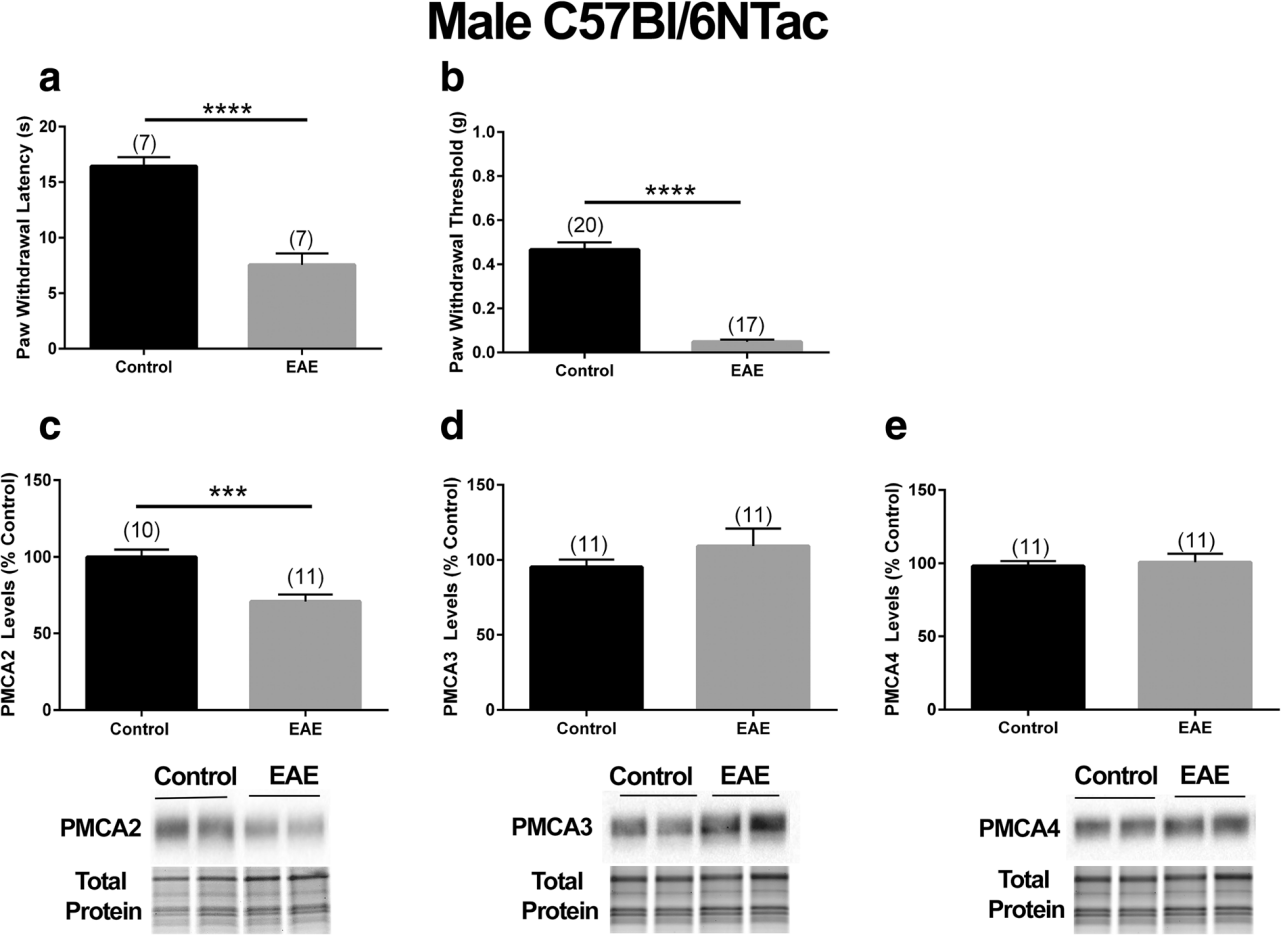
PMCA2 levels in the lumbar DH of male C57Bl/6NTac mice with EAE manifesting pain. Evaluation of thermal (heat) and mechanical pain sensitivity in male mice at early phase of EAE by **a** the Hargreaves test and **b** von Frey filament test. **c**–**e** PMCA2, PMAC3, and PMCA4 protein levels in the lumbar DH. The graphs (upper panel) show the quantification of the band intensity in the Western blots (lower panel; two representative lanes per group). Total protein was used to normalize for experimental variations. The number of mice in each group is shown above bars. Values represent mean ± SEM. Significantly different by independent Student’s *t* test with ***p < 0.001 and ****p < 0.0001

To determine whether the decrease in PMCA2 and increase in pain sensitivity were sex-biased, we induced EAE in male C57Bl/6NTac mice and performed behavioral analysis when the mice experienced only a flaccid tail, as described above. We found a 56% decrease in paw withdrawal latency in the Hargreaves test (Fig. 3a) and an 87% decrease in the paw withdrawal thresholds in the von Frey filament test (Fig. 3b) in EAE mice compared to controls. Moreover, PMCA2 protein levels were selectively and significantly decreased in these mice (Fig. 3c) while PMCA3 and PMCA4 levels remained unchanged (Fig. 3d and e).

### PMCA2 levels are not decreased in the DH of mice that do not manifest pain during EAE

**Fig. 4:**
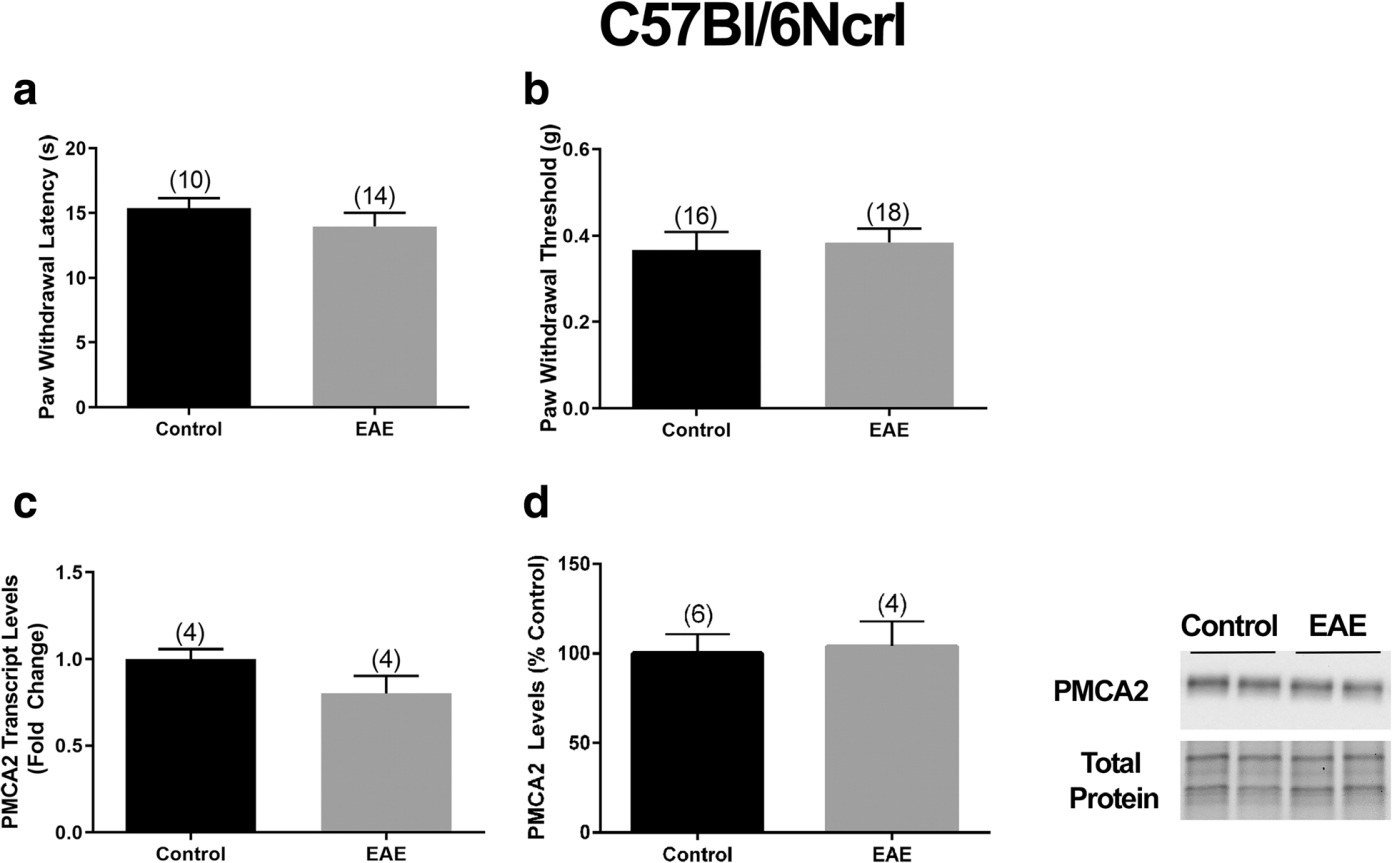
PMCA2 levels in the lumbar DH of C57Bl/6Ncrl mice with EAE, not manifesting pain. Assessment of the **a** Hargreaves test and **b** von Frey filament test in mice with EAE at early phase of the disease. **c** PMCA2 transcript levels in the lumbar DH. **d** PMCA2 protein levels in the lumbar DH. The right panel shows the Western blot (two representative lanes per group), and the graphs in the left panels show the quantification of the band intensity in the Western blot. The number of mice in each group is shown above bars. Values represent mean ± SEM. There were no significant differences by independent Student’s *t* test

Our next goal was to demonstrate that a decrease in PMCA2 in the DH of mice with EAE occurs only when the mice display increased pain sensitivity. The strategy that we used to achieve this goal was to induce EAE in a C57Bl/6N mouse substrain that would develop motor deficits comparable to those observed in the C57Bl/6NTac mice, but would not present with increased pain sensitivity at early disease phase. We postulated that if a decrease in PMCA2 in the DH was indeed associated with pain, there would be no changes in PMCA2 levels in mice showing only motor deficits without pain symptoms. The rationale behind this postulate was based on previous reports showing that diverse mouse strains and C57Bl/6N mouse substrains exhibit genetic differences and phenotypic dissimilarities in physiological, metabolic, immunological, and behavioral responses [32–35]. Indeed, a C57Bl/6N substrain obtained from a different vendor (C57Bl/6Ncrl) displayed motor deficits with no signs of pain at early phase of EAE. The clinical scores at early disease phase were comparable in C57Bl/6Ncrl and C57Bl/6NTac mice (Additional file 1). In both strains, mice inoculated with MOG_35–55_ displayed a flaccid tail (clinical score 1) on day 13 post-inoculation. Despite motor dysfunction, the C57Bl/6Ncrl mice did not show increased sensitivity to heat-induced or mechanical pain at this stage of the disease (Fig. 4a, b). Accordingly, PMCA2 transcript or protein expression in the DH of C57Bl/6Ncrl mice with EAE was not reduced and no statistical differences were observed in PMCA2 levels in the MOG_35–55_ and CFA-inoculated C57Bl/6Ncrl mice (Fig. 4c, d). It is important to note that EAE was induced in C57Bl/6Ncrl and C57Bl/6NTac mice in the same experiment by utilizing the same preparation of MOG_35–55_ and CFA emulsion and behavioral assessments were performed side-by-side, to enable comparison. These results indicated that PMCA2 levels in the DH are not altered when mice inoculated with MOG_35–55_ exhibit motor deficits but do not display increased pain. The findings lend further support to the notion of a coincidence between a decrease in PMCA2 and manifestation of pain during EAE.

### Inflammatory reaction and cytokine expression in the DH of C57Bl/6NTac and C57Bl/6Ncrl mice during EAE

A hallmark of EAE is SC inflammation [36–38]. Because our studies specially focused on the DH, we undertook investigations to characterize the cellular and molecular inflammatory changes in the isolated lumbar DH of the C57Bl/6NTac mice at an early phase of EAE.

**Fig. 5:**
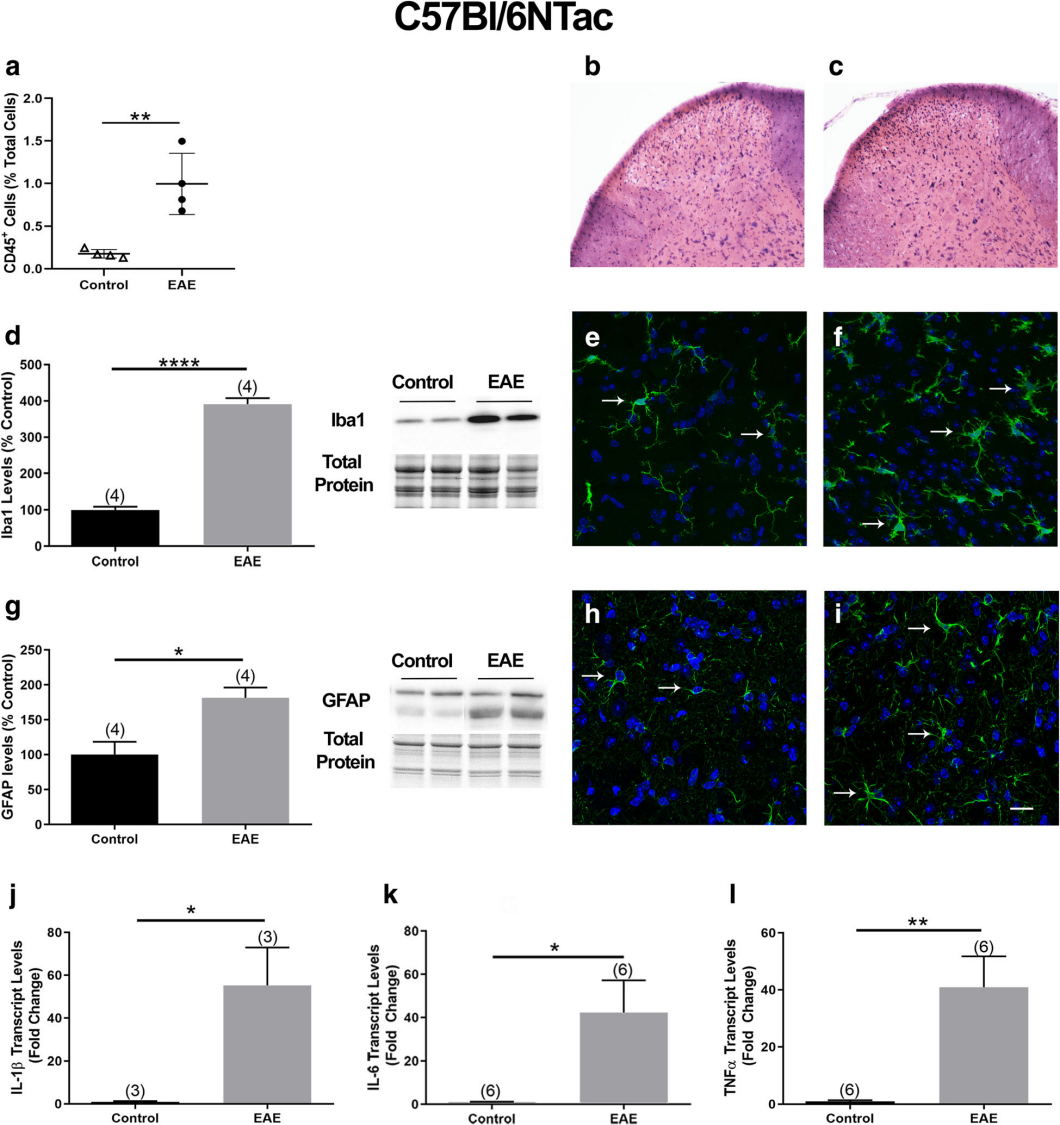
Inflammatory reaction in the DH of C57Bl/6NTac mice with EAE. Assessment of **a** the percentage of CD45^+^ cells in the DH of C57Bl/6NTac mice, analyzed by flow cytometry. **b** and **c** Representative images of H&E staining in the lumbar DH of a control and EAE mouse, respectively. **d** Iba1 protein levels in the lumbar DH of mice with EAE. The graphs (left panel) show the quantification of the band intensity in the Western blots (right panel; two representative lanes per group). Total protein was used to normalize for experimental variations. **e** and **f** Representative images of the DH in a transverse lumbar SC section of a control and EAE mouse, respectively, showing Iba1 immunopositive cells (green) counterstained with DAPI (blue). **g** GFAP protein levels in the lumbar DH of mice with EAE. The graphs (left panel) show the quantification of the band intensity in the Western blots (right panel; two representative lanes per group). Total protein was used to normalize for experimental variations. **h** and **i** Representative images of GFAP (green) and DAPI (blue) double-labeled cells in the DH of a transverse lumbar SC section of a control and EAE mouse, respectively. Arrows point at examples of Iba1 or GFAP-labeled cells. Scale bar represents 10 μm. **j**–**l** IL-1β, IL-6, and TNFα transcript levels in the lumbar DH . The number of mice in each group is shown above bars. Values represent mean ± SEM. Significantly different by independent Student’s *t* test with *p < 0.05, **p < 0.01, and ****p < 0.0001

The percentage of CD45^+^ cells was determined by flow cytometry in the lumbar DH of C57Bl/6NTac mice inoculated with MOG_35–55_ and manifesting both a flaccid tail and increased pain sensitivity. DHs obtained from CFA-inoculated mice were used as controls. In CFA controls, about 0.17% of total cells were CD45^+^, while the percentage of CD45^+^ cells in mice with EAE was significantly higher and was 1% of the total cells (Fig. 5a). Differential infiltration of cells into the lumbar DH was also observed by H&E staining in control mice (Fig. 5b) versus mice with EAE (Fig. 5c).

We next analyzed the protein levels of Iba1, a macrophage/microglia marker, and GFAP, an astrocyte marker. Increased Iba1 has been used as an indicator of microglial activation [39], whereas elevated GFAP has been associated with reactive astrocytes [40]. Western blot analysis demonstrated a significant 4-fold increase in Iba1 levels (Fig. 5d), suggesting microglial activation. This finding was further supported by immunohistochemistry on transverse SC sections of control and EAE mice using an antibody against Iba1 (Fig. 5e, f). GFAP levels were also significantly higher by 56% in the DH of mice with EAE compared to CFA controls (Fig. 5g), suggesting the presence of reactive astrocytes. This idea was further supported by immunohistochemical studies on SC sections by use of an antibody against GFAP (Fig. 5h, i). We also assessed demyelination since it is a hallmark of EAE. Luxol fast blue staining did not reveal demyelination at this stage of EAE (Additional file 2). In addition, we analyzed the expression of three cytokines, IL-1β, IL-6, and TNFα, which have been implicated in pain mechanisms [41, 42] and in the hyperactivation of neurons in the DH [43]. There was a robust 55-, 26-, and 56-fold increase in IL-1β, TNFα, and IL-6 transcript levels, respectively, in the DH of mice with EAE compared to controls (Fig. 5j–l).

**Fig. 6:**
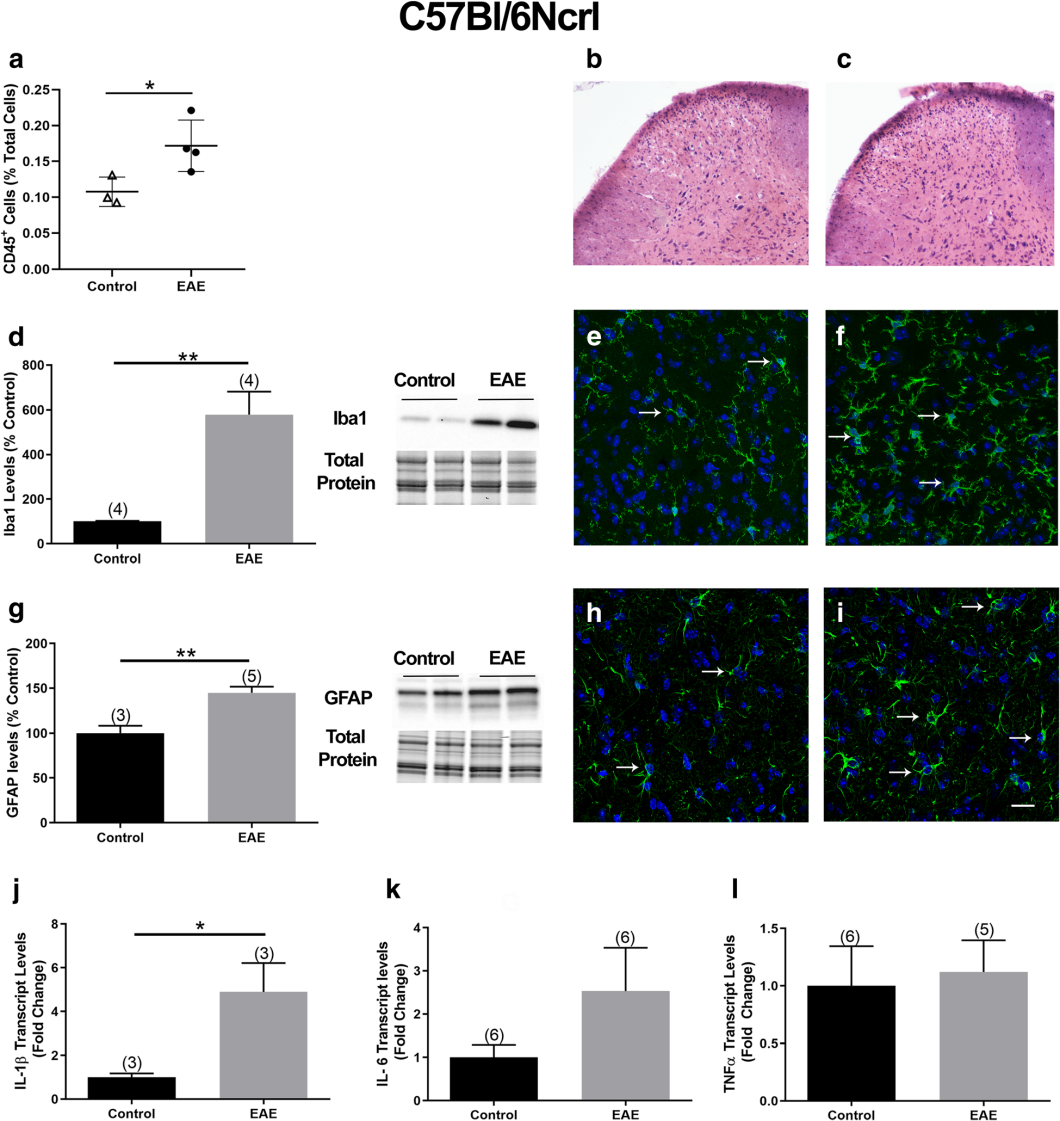
Inflammatory reaction in the DH of C57Bl/6Ncrl mice with EAE. **a** The percentage of CD45^+^ cells in the lumbar DH of C57Bl/6Ncrl mice quantified by flow cytometry. **b** and **c** Representative images showing H&E staining in the lumbar DH of a C57Bl/6Ncrl control and EAE mouse, respectively. **d** Iba1 protein levels in the lumbar DH. The graph (left panel) shows the quantification of the band intensity in the Western blots (right panel; two representative lanes per group). Total protein was used to normalize for experimental variations. **e** and **f** Representative images of the DH in a transverse section of the lumbar SC in a control and EAE mouse, respectively, showing Iba1 immunoreactive cells (green) double-labeled with DAPI (blue). **g** GFAP protein levels in the lumbar DH of mice with EAE. The graph (left panel) shows the quantification of the band intensity in the Western blots (right panel; two representative lanes per group). Total protein was used to normalize for experimental variations. **h **and **i** Representative images of a transverse lumbar SC section showing GFAP (green) and DAPI (blue) double-labeled cells in the DH of a control and EAE mouse, respectively. Arrows point at examples of Iba1 or GFAP-labeled cells. Scale bar represents 10 μm. **j**–**i** IL-1β, IL-6, and TNFα transcript levels in the lumbar DH. The number of mice in each group is shown above bars. Values represent mean ± SEM. Significantly different by independent Student’s *t* test with *p < 0.05 and **p < 0.01

We also undertook investigations to study the same parameters in the C57Bl/6Ncrl mice. Quantification of CD45^+^ cells in the DH of CFA controls and mice with EAE indicated a modest but significant increase in the percentage of CD45^+^ cells (CFA 0.10% and EAE 0.15% of total cells; Fig. 6a). Differential infiltration of cells into the lumbar DH was observed by H&E staining in control mice (Fig. 6b) versus mice with EAE (Fig. 6c).

Iba1 levels increased by - 5-fold in the DH of EAE mice compared to CFA controls (Fig. 6d) indicating microglial activation. Iba1 immunoreactivity in the lumbar DH further supported the idea of microglial activation (Fig. 6e, f). In addition, GFAP protein levels were 46% higher in the DH of EAE mice (Fig. 6g) compared to controls, indicating the presence of reactive astrocytes. This was further supported by immunohistochemical studies utilizing SC sections and an antibody against GFAP (Fig. 6h, i). At this stage of EAE, we did not observe demyelination in the lumbar SC as demonstrated by Luxol fast blue staining (Additional file 3). In contrast to the C57Bl/6NTac mice which showed a robust 55-fold increase in IL-1β transcript levels, there was a relatively modest 6-fold increase in IL-1β transcript levels in the DH of C57Bl/6Ncrl mice (Fig. 6j). No significant differences in TNFα and IL-6 transcript levels were observed between control mice and mice with EAE (Fig. 6k, l)

### IL-1β selectively reduces PMCA2 expression in SC neurons of C57Bl/6NTac mice, in vitro

**Fig. 7:**
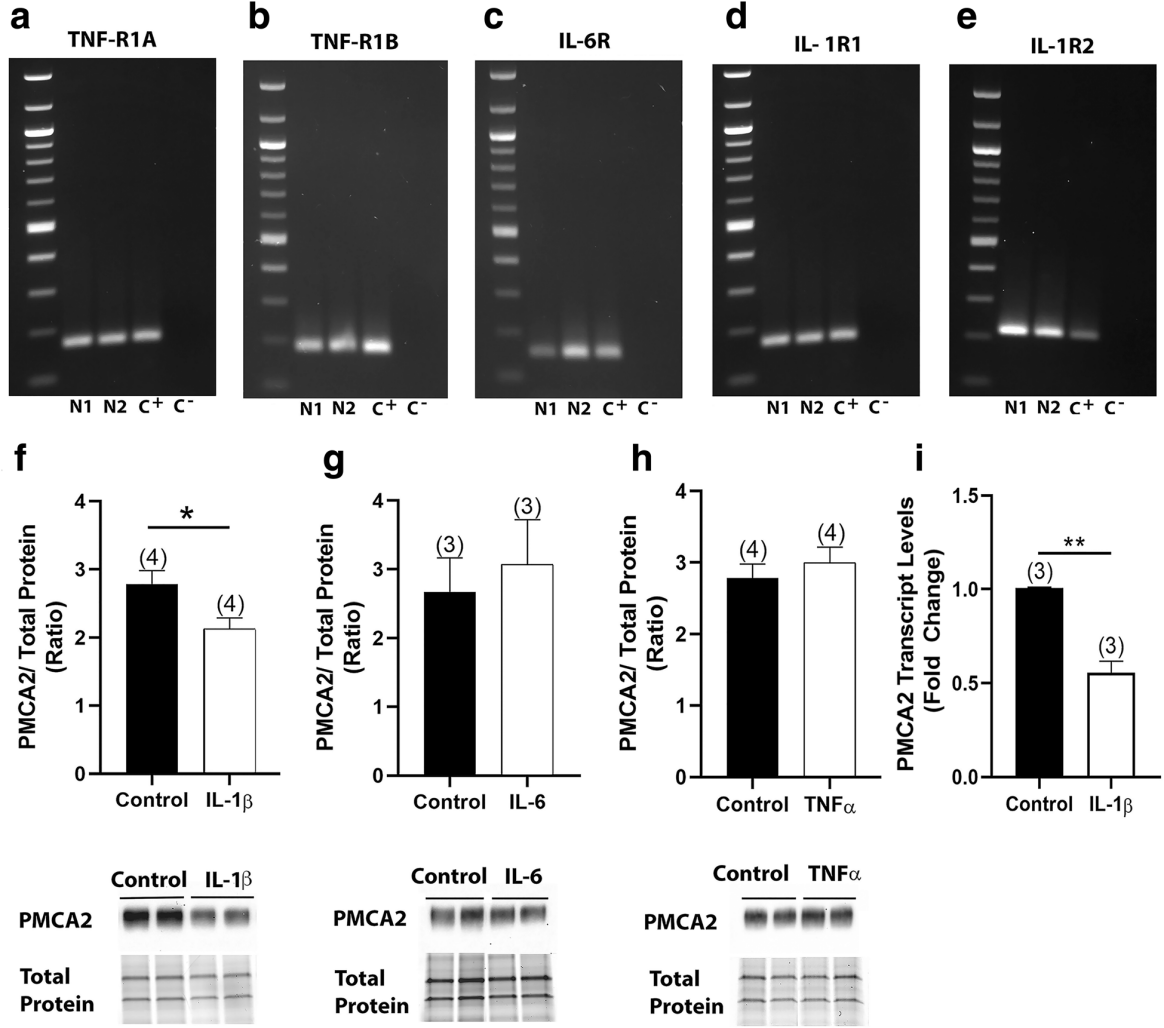
Effects of IL-1β on PMCA2 levels in pure SC neuronal cultures. **a**–**e** Agarose gels showing IL-1β, IL-6, and TNFα receptor transcripts by qRT-PCR in two distinct biological repeats of SC neuronal cultures (N1 and N2). Spleen was used as a positive control (C^+^). Negative control (C^−^) was RNA that was subject to PCR without RT to ensure the lack of contaminating genomic DNA. **f**–**h** Quantification of PMCA2 protein levels in SC neuronal cultures treated with IL-1β, IL-6, or TNFα. Graphs (upper panels) show the quantification of the band intensity in the Western blots (lower panels; two representative lanes per group). Total protein was used to normalize for experimental variations. **i** PMCA2 transcript levels in pure SC neuronal cultures following treatment with IL-1β. Values represent mean ± SEM. Significantly different by independent Student’s *t* test, *p < 0.05 and **p < 0.01

The studies described above determined that the major difference in inflammatory reaction in the lumbar DH of C57Bl/6NTac and C57Bl/6Ncrl mice is the expression of cytokines. Therefore, we postulated that a strong increase in IL-1β, TNFα, and IL-6 could be the reason of a decrease in PMCA2 in the DH of C57Bl/6NTac mice. We determined whether IL-1β, TNFα, and IL-6 are triggers that reduce PMCA2 expression via direct actions on neurons. To address this question, we used pure SC neuronal cultures, which were characterized by our laboratory previously [31]. We first confirmed that transcripts for IL-1β (IL-1R and IL-1R2), IL-6 (IL-6R), and TNFα (TNF-R1A and TNF-R1B) receptors can be detected in neurons using qRT-PCR (Fig. 7a–e). Neuronal cultures treated with IL-1β (10 ng/ml) for 48 h showed a significant 24% decrease in PMCA2 protein levels when compared to sister cultures treated with vehicle (Fig. 7f). In contrast, there were no significant alterations in PMCA2 levels in response to IL-6 (10 ng/ml) or TNFα (10 ng/ml) treatments (Fig. 7g, h). To determine whether the decrease in PMCA2 occurs at the transcriptional level, we used qRT-PCR. There was a 50% decrease in PMCA2 transcript levels in IL-1β-treated neurons compared to vehicle-treated neurons (Fig. 7i). In contrast, IL-1β did not alter PMCA3 protein levels in the same neuronal cultures (Additional file 4). Thus, IL-1β selectively decreases PMCA2 expression in SC neurons. These results together with ex vivo data showing differential expression of IL-1β in the DH of C57Bl/6NTac versus C57Bl/6Ncrl mice during EAE suggest that a robust increase in IL-1β expression might be necessary to reduce PMCA2 expression and to elicit pain responses during EAE, as observed in C57Bl/6NTac mice. In contrast, a modest increase in IL-1β in the DH might not be sufficient to induce a decrease in PMCA2 and elicit increased pain, as observed in the C57Bl/6Ncrl mice with EAE.

In additional studies, we confirmed the involvement of IL-1β in pain responses in C57Bl/6NTac mice with EAE by inhibiting the effects of the cytokine through intrathecal administration of IL-1RA, an IL-1β receptor antagonist. This treatment effectively blocked pain responses as evidenced by the Hargreaves test (Additional file 5).

### Below-level neuropathic pain is paralleled by a decrease in PMCA2 in the lumbar DH of C57Bl/6NTac mice sustaining a mid-thoracic SCI

To provide further evidence for a link between reduced PMCA2 in the DH and pathological pain, we used a mouse model of SC contusion injury. In this model, injury was induced at mid-thoracic (T8) level in C57Bl/6NTac female mice. Below injury pain was evaluated by measuring the paw withdrawal thresholds and latencies at 28 dpi, after mice had spontaneously recovered sufficient hindlimb motor function (Additional file 6) to allow for the performance of the von Frey filament and Hargreaves’ tests. Subsequently, PMCA2 levels were quantified in the lumbar DH, the region receiving pain information from the paws.

**Fig. 8:**
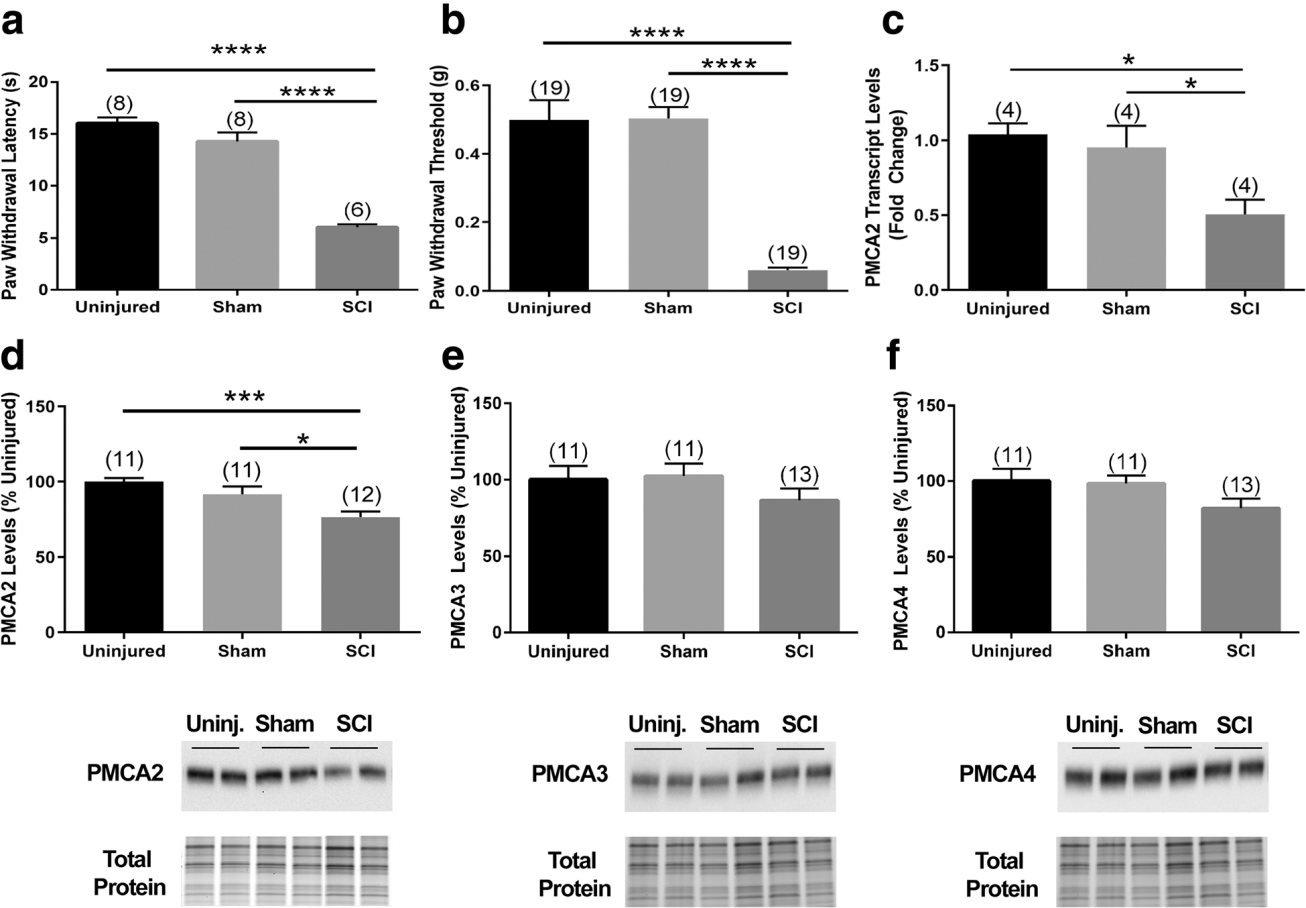
PMCA2 levels in the lumbar DH of mice sustaining a SCI and manifesting chronic pain. Assessment of the **a** Hargreaves test and **b** von Frey filament test in mice sustaining a SCI at 28 dpi. **c** PMCA2 transcript levels in the lumbar DH of mice at 28 dpi. **d** PMCA2, **e** PMAC3, and **f** PMCA4 protein levels in the lumbar DH of mice at 28 dpi. Graphs (upper panels) show the quantification of the band intensity in the Western blots (lower panels; two representative lanes per group). Total protein was used to normalize for experimental variations. The number of mice in each group is shown above bars. Values represent mean ± SEM. Significantly different by one-way ANOVA and Tukey’s post hoc test. *p < 0.05, ***p < 0.001, and ****p < 0.0001

SCI elicited a significant increase in both heat (Fig. 8a) and mechanical sensitivity (Fig. 8b) in injured mice compared to shams and uninjured controls. There was a significant 40% decrease in PMCA2 transcript levels (Fig. 8c) and about 30% reduction in PMCA2 protein levels (Fig. 8d). In contrast, PMCA3 and PMCA4 protein levels did not change in the lumbar DH of mice that sustained a SCI, indicating that the decrease in PMCA2 levels is selective (Fig. 8e, f).

## Discussion

The investigations reported here show, for the first time, that the manifestation of pain in EAE and SCI is paralleled by a decrease in PMCA2 levels in the DH of the SC. In contrast, the levels of two other isoforms, PMCA3 and PMCA4, were not affected by injury or disease, supporting the notion that PMCA2 selectively responds to pathological changes in the DH during EAE or SCI. We also found that IL-1β, which is known to play a critical role in neuropathic pain [44], is a trigger that downregulates PMCA2 expression through direct actions on SC neurons, in vitro. It has previously been reported that IL-1β is increased in the SC during EAE [45], a finding that we validated by analyzing the isolated DH. To our knowledge, this is the first report postulating that PMCA2 contributes to pathological pain mechanisms in the DH during injury or inflammatory disease, and therefore, it is a potential new target for therapeutic interventions.

The present investigations primarily focused on a mouse model of MS because pain is a common symptom of the disease [46] even though the majority of investigations on rodent models of MS focus on motor dysfunction. About 60% of individuals with MS experience pain that can manifest in various forms, whereas approximately 25–40% of subjects with MS are diagnosed with neuropathic pain that is not adequately alleviated by currently available pharmacotherapies [47]. Pain can even affect some individuals with MS at an early disease phase [48]. In agreement with this idea, increased pain sensitivity was already evident at the initial phase of the disease in C57Bl/6NTac mice, at the time when only a flaccid tail was observed. Taking into consideration the fact that pain is a symptom observed in some, but not all individuals with MS, we attempted to replicate the human condition by using a different C57Bl/6N mouse substrain, the C57Bl/6Ncrl mice, which developed motor symptoms comparable to those displayed by the C57Bl/6NTac mice, but diverged in the manifestation of pain. Accordingly, PMCA2 levels in the DH were not altered in the C57Bl/6Ncrl mice that manifested only motor symptoms during the early phase of EAE. As indicated by several studies, genetic variances and phenotypic differences in metabolic, immunological, and behavioral responses among mouse strains [49] and substrains [50] have been observed. Such differences have been particularly well-delineated in the C57Bl/6N substrain [32, 33]. Therefore, it is not surprising that the C57Bl/6NTac and C57Bl/6Ncrl show dissimilar pain responses even though the development of motor symptoms is comparable and quadriplegia (clinical score 4) occurs within the same period of time post-inoculation. However, it is worth noting that we examined pain sensitivity in both C57Bl/6Ncrl and C57Bl/6NTac mice only at the initial phase of EAE because hindlimb paresis and paralysis at later disease phases interfere with the reliable execution of assays that measure pain responses. Therefore, we cannot rule out the possibility that the C57Bl/6Ncrl mice ultimately develop increased pain sensitivity at a more advanced stage of EAE. Nonetheless, taken together, these results conceptually support a link between decreased PMCA2 expression in the DH and increased sensitivity to pain during EAE. This notion is further reinforced by the fact that in a model of SC contusion injury, a pathological condition that is also associated with chronic pain [2], increased mechanical and heat sensitivity below the level of injury (in the hindlimbs) coincides with a concomitant decrease in PMCA2 in the lumbar DH, a region remote from the injury epicenter that receives pain information from the hindlimbs.

In the present study, both male and female C57Bl/6NTac mice showed increased pain sensitivity during EAE which was paralleled by a decrease in PMCA2 levels, suggesting that the involvement of PMCA2 in pain responses in inflammatory disease does not show sex bias. This is in agreement with the finding that the incidence of reported chronic pain in male and female patients with MS is similar [4], increasing the clinical significance of our findings. Interestingly, in earlier investigations that utilized healthy, whole body mutant PMCA2^+/−^ mice, we showed that female PMCA2^+/−^ mice are more sensitive to evoked mechanical pain than wild-type controls of the same sex, whereas such a difference was not observed in male PMCA2^+/−^ and PMCA2^+/+^ mice [13]. These apparently discordant results can be explained in various ways. The studies of Khariv et al. focused on nociceptive pain in healthy mutant mice whereas the present investigations analyzed inflammatory disease-induced neuropathic pain in wild-type mice. The involvement of PMCA2 in nociceptive versus neuropathic pain in female as opposed to male mice could be different. Alternatively, in PMCA2^+/−^ mice, PMCA2 levels are lower throughout embryonic and postnatal development and this could differently affect the development and establishment of pain circuits in the SC of female versus male mice. In contrast, in the wild-type adult mice that were used in the present study, these circuits had already been fully established in male and female mice under circumstances of normal PMCA2 expression levels.

What are the triggers that cause a reduction in PMCA2 in the DH of mice with EAE manifesting pain? To address this issue, we focused on cytokines because a hallmark of EAE is SC inflammation, infiltration of immune cells [51], and glial activation [52]. The strongest inflammatory reaction occurs in the lumbar region, whereas inflammation is less pronounced, but still present, in the thoracic and cervical SC [53]. Microglia and astrocyte activation also contribute to EAE pathology [52]. Importantly, activated glia have been implicated in chronic pain mechanisms in the DH [54, 55]. Therefore, we hypothesized that soluble factors released by infiltrating immune cells and/or activated glia act on neurons and cause a reduction in PMCA2 expression in the DH of mice with EAE. As soluble factors, we considered cytokines because they play important roles in pain mechanisms in the DH. We focused especially on IL-1β, IL-6, and TNFα because they have often been implicated in pain mechanisms [56]. These cytokines are upregulated in various injury models, and blocking their respective receptors improves or prevents inflammatory hyperalgesia or nerve injury-induced mechanical allodynia [57]. IL-1β, IL-6, and TNFα also play important roles in the sensitization of neurons mediating pain transmission in the DH. They alter neuronal activity by modulating excitatory and inhibitory neurotransmission in the DH, which could lead to pain [43].

Both activated glia and infiltrating immune cells release IL-1β, IL-6, and TNFα [58, 59]. Even though the expression of all three cytokines was robustly increased in the DH of the C57Bl/6NTac mice during EAE and albeit expression of receptors for all three cytokines in SC neurons, in vitro, only IL-1β suppressed PMCA2 levels in SC neuronal cultures. This suggests that downregulation of PMCA2 expression in the DH is not a generic outcome of inflammation and depends selectively on IL-1β. Interestingly, in the DH of C57Bl/6Ncrl mice, there was only a modest increase in IL-1β expression. Since PMCA2 levels in the DH of these mice were not decreased, we propose that downregulation of PMCA2 in DH neurons necessitates high IL-1β expression. Moreover, even though IL-6 and TNFα did not decrease PMCA2 in SC neuronal cultures through direct actions, they could have indirect effects on PMCA2 expression in vivo, via actions on other cell types. In C57Bl/6NTac mice, IL-6 and TNFα levels in the DH were robustly increased during EAE, whereas in C57Bl/6Ncrl, they were not altered. This also could have contributed to the differential pain responses and PMCA2 expression in the two substrains during EAE.

The reduction in PMCA2 levels in the lumbar DH of mice sustaining a SCI could be the result of cellular and molecular changes that are different than those occurring in EAE because in the SCI model, infiltrating immune cells are not present in the lumbar SC [60]. The lumbar region is considerably remote from the lesion site where infiltrating immune cells are abundant [60]. However, persistent microglial activation is observed in the lumbar SC at 28 dpi, a sub-acute SCI phase during which we evaluated both pain and PMCA2 protein levels. Therefore, it is likely that the effectors that reduce PMCA2 expression in the lumbar DH are secreted by activated microglia following SCI and these might be different from the triggers responsible for the decrease in PMCA2 during EAE. In agreement with this idea, we did not find a significant increase in IL-1β transcripts in the lumbar DH at 28 dpi (Additional file 7). Nevertheless, the reduction in PMCA2 might be relevant to pain mechanisms in both models, irrespective of the trigger that downregulates PMCA2.

**Fig. 9:**
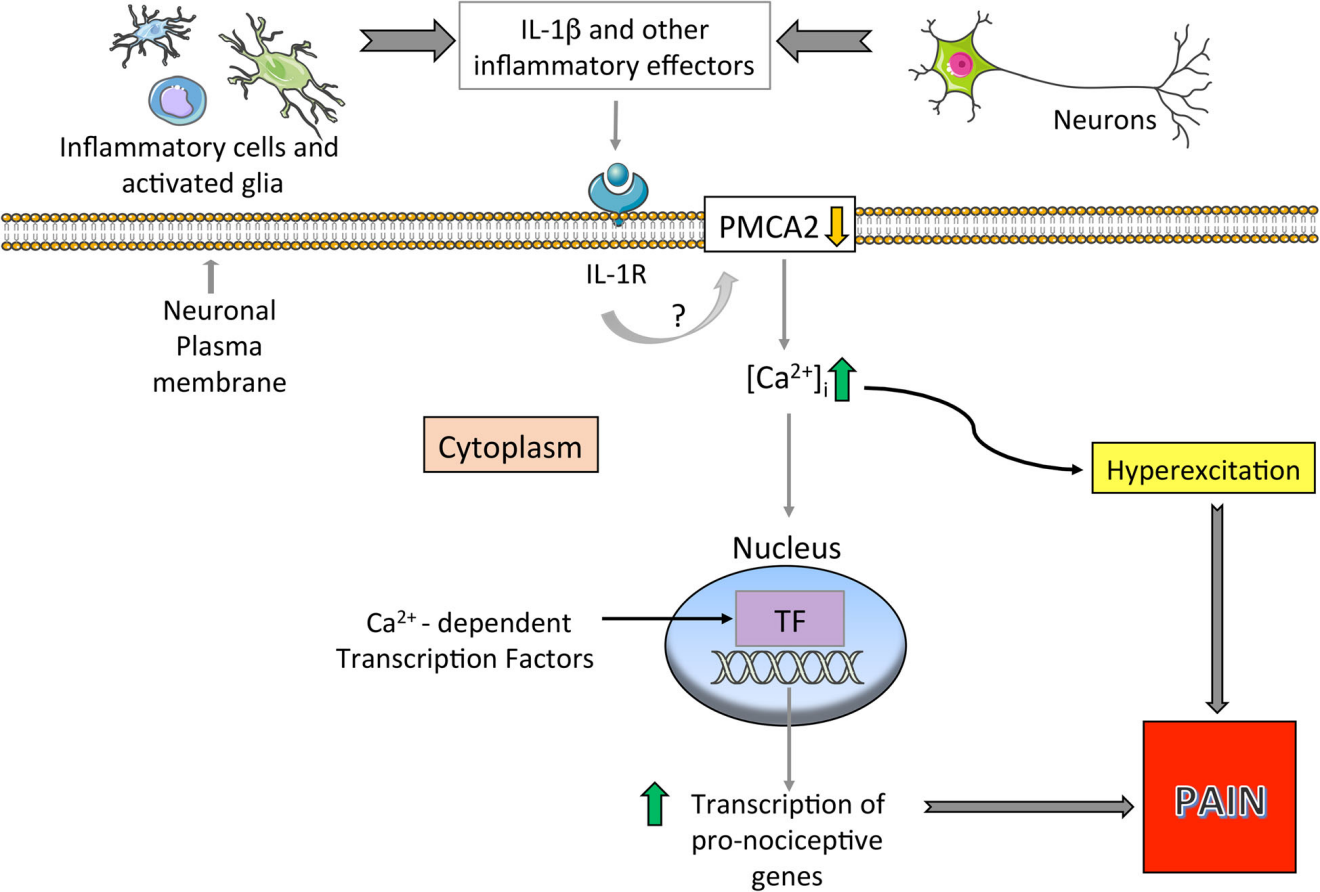
Schematic summary of the proposed pain mechanism in the DH of mice with EAE. The inflammatory milieu is the source of triggers, including IL-1β, which decreases PMCA2 expression. The reduction in PMCA2 leads to increased intracellular Ca^2+^ [Ca^2+^]_i_. This, in turn, enhances the hyperexcitation of neurons and promotes the activation of Ca^2+^-dependent transcription factors which support the expression of pro-nociceptive genes. The total outcome of hyperexcitation and increased pro-nociceptive gene transcription is induction of pain

The mechanisms by which PMCA2 contributes to pain processing remain undefined. We have previously shown that the amplitude of Ca^2+^ transients is increased and the clearance of Ca^2+^ transients is delayed when PMCA activity is inhibited in SC neurons, in vitro [61]. Since cytoplasmic Ca^2+^ can regulate the excitability of neurons [62, 63], the persistence of high intracellular Ca^2+^ following influx could lead to hyperexcitation of DH neurons and promote abnormal neuronal function due to perturbations in Ca^2+^-dependent intracellular signaling and gene transcription. This proposed mechanism is illustrated in Fig. 9.

Voltage-gated calcium channels play essential roles in regulating the excitability of DH neurons and interneurons which process the pain information received from the DRG and transmitted to the brain [64]. This highlights the central contribution of Ca^2+^ signaling to pain mechanisms in the DH. Despite the critical role played by calcium pumps and exchangers in neuronal function in health and disease [65, 66], their contribution to pain information processing has not been adequately investigated. Yet, several lines of evidence indicate that they might be key contributors to this process. In DRG-SC neuronal co-cultures, blocking PMCA activity slowed presynaptic Ca^2+^ clearance and signaling in DRG neurons [67] whereas inhibition of other calcium pumps and exchangers did not have major effects. In a spinal nerve ligation model, sarco-endoplasmic reticulum Ca^+ 2^-ATPase (SERCA) activity was decreased in axotomized small sensory neurons suggesting that loss of SERCA function contributes to the generation of pain following peripheral nerve injury [68]. In a similar model of nerve injury, PMCA function in axotomized DRG neurons was increased and the afterhyperpolarization (AHP), a regulator of neuronal excitability [69], was prolonged [70]. Of note, the majority of the studies on PMCA function in sensory neurons of the DRG did not differentiate between isoforms because the inhibitors used in these investigations block the activity of all isoforms. In contrast, our present studies focused on PMCA isoform 2 in DH neurons and therefore provide a new and distinct perspective.

## Conclusions

The present studies establish a link between PMCA2 expression in the DH and increased pain sensitivity in animal models of MS and SCI. It is proposed that PMCA2 plays a role in pain processing in the DH by inducing changes in intracellular Ca^2+^, which can affect the hyperexcitability of DH neurons and increase pro-nociceptive gene transcription. Effectors released by inflammatory cells and activated glia and, in particular, IL-1β could trigger a reduction in PMCA2 levels in DH neurons. It is worth noting that we have previously reported that PMCA2 is also decreased in the ventral horn motor neurons during EAE [24, 71] and that the decrease in PMCA2 increases the vulnerability of motor neurons to injury and death [61, 72]. Thus, restoration of PMCA2 in the SC could alleviate not only pain but also motor deficits during EAE.

## Supplementary information

**Additional file 1.** Clinical scores at early EAE phase are comparable in C57Bl/6Ncrl and C57Bl/6NTac mice. Clinical scores were evaluated in C57Bl/6NTac and C57Bl/6Ncrl mice side-by-side, as described in Methods. The graph shows the scores at early disease phase. Mice were evaluated for pain when they manifested flaccid tail (clinical score 1).

**Additional file 2.** Absence of demyelination in the lumbar SC of C57Bl/6NTac mice with EAE at early disease phase. Lumbar SC sections, obtained from A) CFA- or B) MOG_35–55-_inoculated C57Bl/6NTac mice manifesting only flaccid tail, were stained with Luxol fast blue and revealed no demyelination.

**Additional file 3.** Absence of demyelination in the lumbar SC of C57Bl/6Ncrl mice with EAE at early disease phase. Lumbar SC sections, obtained from A) CFA- or B) MOG_35–55-_inoculated C57Bl/6Ncrl mice manifesting only flaccid tail, were stained with Luxol fast blue and revealed no demyelination.

**Additional file 4.** PMCA3 protein levels in neuronal cultures following IL-1β treatment. The graph (left panel) shows the quantification of the band intensity in the western blot (right panel; 2 representative lanes per group). Total protein was used to normalize for experimental variations. Values represent mean ± SEM. There were no significant differences by independent Student’s t-test.

**Additional file 5.** Intrathecal IL-1RA treatment ameliorates pain in C57Bl/6NTac mice during EAE. Evaluation of thermal (heat) pain sensitivity in female C57Bl/6NTac mice with EAE following IL-1RA treatment. Vehicle or IL-1RA was administered by lumbar puncture to control mice and mice with EAE when the first motor symptom (weakness of only the tip of the tail) was observed. This was followed by a second intrathecal injection 24 h later. Pain was assessed when mice developed flaccid tail (clinical score 1). Intrathecal IL-1RA significantly ameliorated pain as indicated by the restoration of paw withdrawal latencies to control values.

**Additional file 6.** Open field locomotor function in C57Bl/6NTac mice following SCI. Open field locomotor function in female mice that sustained a mid-thoracic contusion injury was assessed using the BMS scale on 1, 7, 14, 21 and 28 dpi. Locomotor function in sham and uninjured mice was also assessed concomitantly.

**Additional file 7.** IL-1β levels are unaltered in the lumbar DH of C57Bl/6NTac mice following SCI. Graph showing IL-1β transcript levels in the lumbar DH of female C57Bl/6NTac mice at 28 dpi. The number of mice in each group is shown above bars. Values represent mean ± SEM. There were no significant differences by one-way ANOVA.

## Data Availability

The datasets used and/or analyzed during the current study are available from the corresponding author on reasonable request.

## Abbreviations

CFA: Complete Freund’s Adjuvant
CNS: Central nervous system
DH: Dorsal horn
DPI: Days post-injury
DRG: Dorsal root ganglia
EAE: Experimental autoimmune encephalomyelitis
GFAP: Glial fibrillary acidic protein
Iba1: Ionized calcium binding adaptor molecule 1
IL-1β: Interleukin-1β
IL-6: Interleukin-6
MOG_35–55_: Myelin oligodendrocyte glycoprotein fragment 35–55
MS: Multiple sclerosis
NeuN: Neuronal nuclei
PMCA2: Plasma membrane calcium ATPase 2
PMCA3: Plasma membrane calcium ATPase 3
PMCA4: Plasma membrane calcium ATPase 4
SC: Spinal cord
SCI: Spinal cord injury
TNFα: Tumor necrosis factor α
