# Supplementary material for: Pathological pain processing in mouse models of multiple sclerosis and spinal cord injury: contribution of plasma membrane calcium ATPase 2 (PMCA2)
Source: J Neuroinflammation. 2019 Nov 8;16:207. doi: 10.1186/s12974-019-1585-2 (PMC6839084; doi:10.1186/s12974-019-1585-2)
Supplement: Supplementary file 1 — Additional file 1. Clinical scores at early EAE phase are comparable in C57Bl/6Ncrl and C57Bl/6NTac mice. Clinical scores were evaluated in C57Bl/6NTac and C57Bl/6Ncrl mice side-by-side, as described in Methods. The graph shows the scores at early disease phase. Mice were evaluated for pain when they manifested flaccid tail (clinical score 1). [file 12974_2019_1585_MOESM1_ESM.pdf]

Additional Files

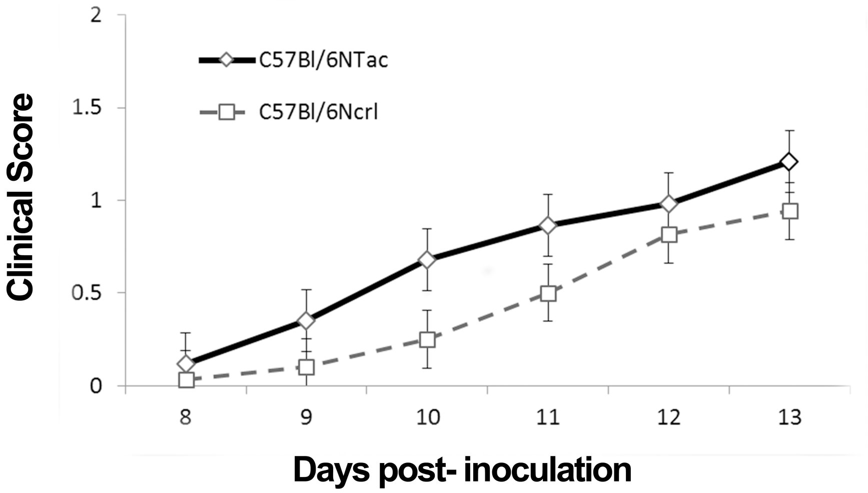

Additional file 1. Clinical scores at early EAE phase are comparable in C57Bl/6NTac and C57Bl/6Ncrl mice

Clinical scores were evaluated in C57Bl/6NTac and C57Bl/6Ncrl mice side-by-side, as described in Methods. The graph shows the scores at early disease phase. Mice were evaluated for pain when they manifested flaccid tail (clinical score 1).
