# Supplementary material for: Pathological pain processing in mouse models of multiple sclerosis and spinal cord injury: contribution of plasma membrane calcium ATPase 2 (PMCA2)
Source: J Neuroinflammation. 2019 Nov 8;16:207. doi: 10.1186/s12974-019-1585-2 (PMC6839084; doi:10.1186/s12974-019-1585-2)
Supplement: Supplementary file 2 — Additional file 2. Absence of demyelination in the lumbar SC of C57Bl/6NTac mice with EAE at early disease phase. Lumbar SC sections, obtained from A) CFA- or B) MOG35–55-inoculated C57Bl/6NTac mice manifesting only flaccid tail, were stained with Luxol fast blue and revealed no demyelination. [file 12974_2019_1585_MOESM2_ESM.pdf]

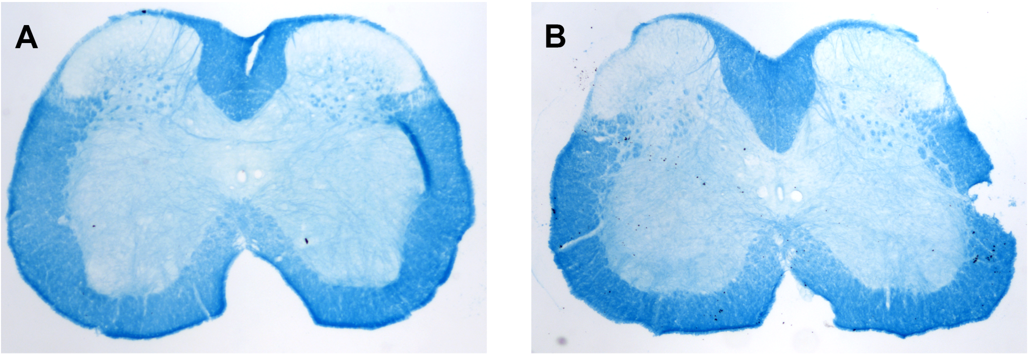

Additional file 2. Absence of demyelination in the lumbar SC of C57Bl/6NTac mice with EAE at early disease phase.

Lumbar SC sections, obtained from A) CFA- or B) MOG_{35-55}-inoculated C57Bl/6NTac mice manifesting only flaccid tail, were stained with Luxol fast blue and revealed no demyelination.
