# Supplementary material for: Pathological pain processing in mouse models of multiple sclerosis and spinal cord injury: contribution of plasma membrane calcium ATPase 2 (PMCA2)
Source: J Neuroinflammation. 2019 Nov 8;16:207. doi: 10.1186/s12974-019-1585-2 (PMC6839084; doi:10.1186/s12974-019-1585-2)
Supplement: Supplementary file 4 — Additional file 4. PMCA3 protein levels in neuronal cultures following IL-1β treatment. The graph (left panel) shows the quantification of the band intensity in the western blot (right panel; 2 representative lanes per group). Total protein was used to normalize for experimental variations. Values represent mean ± SEM. There were no significant differences by independent Student’s t-test. [file 12974_2019_1585_MOESM4_ESM.pdf]

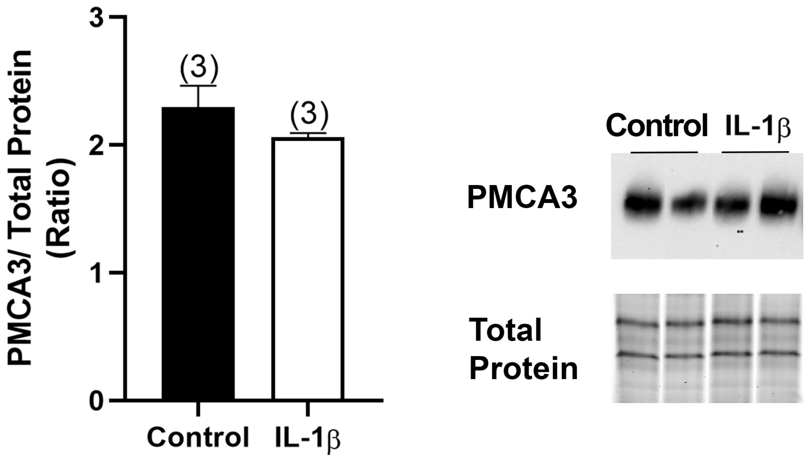

Additional file 4. PMCA3 protein levels in neuronal cultures following IL-1β treatment.

The graph (left panel) shows the quantification of the band intensity in the western blot (right panel; 2 representative lanes per group). Total protein was used to normalize for experimental variations. Values represent mean ± SEM. There were no significant differences by independent Student's t-test.
