# Supplementary material for: Pathological pain processing in mouse models of multiple sclerosis and spinal cord injury: contribution of plasma membrane calcium ATPase 2 (PMCA2)
Source: J Neuroinflammation. 2019 Nov 8;16:207. doi: 10.1186/s12974-019-1585-2 (PMC6839084; doi:10.1186/s12974-019-1585-2)
Supplement: Supplementary file 5 — Additional file 5. Intrathecal IL-1RA treatment ameliorates pain in C57Bl/6NTac mice during EAE. Evaluation of thermal (heat) pain sensitivity in female C57Bl/6NTac mice with EAE following IL-1RA treatment. Vehicle or IL-1RA was administered by lumbar puncture to control mice and mice with EAE when the first motor symptom (weakness of only the tip of the tail) was observed. This was followed by a second intrathecal injection 24 h later. Pain was assessed when mice developed flaccid tail (clinical score 1). Intrathecal IL-1RA significantly ameliorated pain as indicated by the restoration of paw withdrawal latencies to control values. [file 12974_2019_1585_MOESM5_ESM.pdf]

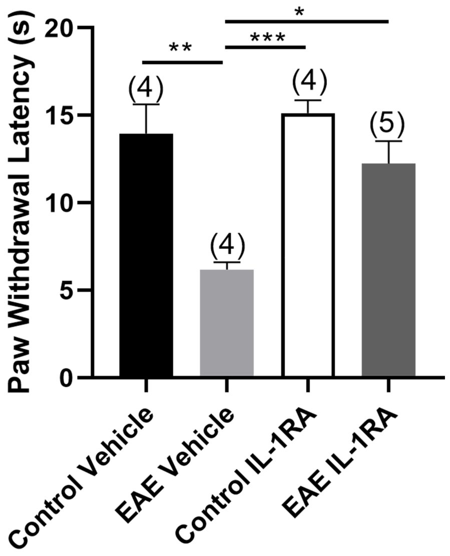

Additional file 5. Intrathecal IL-1RA treatment ameliorates pain in C57Bl/6NTac mice during EAE.

Evaluation of thermal (heat) pain sensitivity in female C57Bl/6NTac mice with EAE following IL-1RA treatment. Vehicle or IL-1RA was administered by lumbar puncture to control mice and mice with EAE when the first motor symptom (weakness of only the tip of the tail) was observed. This was followed by a second intrathecal injection 24 hrs later. Pain was assessed when mice developed flaccid tail (clinical score 1). Intrathecal IL-1RA significantly ameliorated pain as indicated by the restoration of paw withdrawal latencies to control values.
