# Supplementary material for: Pathological pain processing in mouse models of multiple sclerosis and spinal cord injury: contribution of plasma membrane calcium ATPase 2 (PMCA2)
Source: J Neuroinflammation. 2019 Nov 8;16:207. doi: 10.1186/s12974-019-1585-2 (PMC6839084; doi:10.1186/s12974-019-1585-2)
Supplement: Supplementary file 6 — Additional file 6. Open field locomotor function in C57Bl/6NTac mice following SCI. Open field locomotor function in female mice that sustained a mid-thoracic contusion injury was assessed using the BMS scale on 1, 7, 14, 21 and 28 dpi. Locomotor function in sham and uninjured mice was also assessed concomitantly. [file 12974_2019_1585_MOESM6_ESM.pdf]

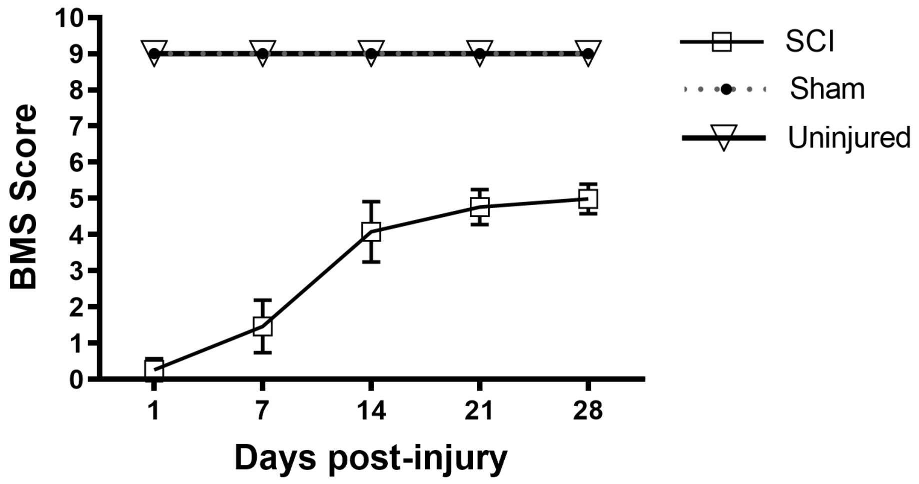

Additional file 6. Open field locomotor function in C57Bl/6NTac mice following SCI.

Open field locomotor function in female mice that sustained a mid-thoracic contusion injury was assessed using the BMS scale on 1, 7, 14, 21 and 28 dpi. Locomotor function in sham and uninjured mice was also assessed concomitantly.
