# Supplementary material for: Pathological pain processing in mouse models of multiple sclerosis and spinal cord injury: contribution of plasma membrane calcium ATPase 2 (PMCA2)
Source: J Neuroinflammation. 2019 Nov 8;16:207. doi: 10.1186/s12974-019-1585-2 (PMC6839084; doi:10.1186/s12974-019-1585-2)
Supplement: Supplementary file 7 — Additional file 7. IL-1β levels are unaltered in the lumbar DH of C57Bl/6NTac mice following SCI. Graph showing IL-1β transcript levels in the lumbar DH of female C57Bl/6NTac mice at 28 dpi. The number of mice in each group is shown above bars. Values represent mean ± SEM. There were no significant differences by one-way ANOVA. [file 12974_2019_1585_MOESM7_ESM.pdf]

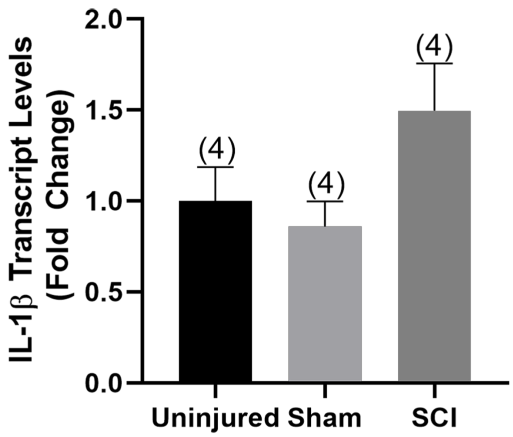

Additional file 7. IL-1β levels are unaltered in the lumbar DH of C57Bl/6NTac mice following SCI

Graph showing IL-1β transcript levels in the lumbar DH of female C57Bl/6NTac mice at 28 dpi. The number of mice in each group is shown above bars. Values represent mean $\pm$ SEM. There were no significant differences by one-way ANOVA.
